# The type of COVID−19 vaccination does not affect reproductive function and pregnancy outcomes in infertile couples

**DOI:** 10.3389/fendo.2024.1356938

**Published:** 2024-06-14

**Authors:** Shiming Wang, Ningning Wang, Guidong Yao, Yingchun Su, Lin Qi

**Affiliations:** Reproductive Medical Center, The First Affiliated Hospital of Zhengzhou University, Zhengzhou, China

**Keywords:** infertile couple, IVF, pregnancy rate, COVID-19 vaccine, cohort study or case-control study

## Abstract

**Introduction:**

Studies on the effect of vaccine type and two other vaccines other than inactivated vaccines approved in China on *in vitro* fertilization (IVF) pregnancy outcomes are rare. To complement and confirm the existing findings, this research aimed to investigate whether there are adverse effects of different vaccine types in females and males on reproductive function and clinical pregnancy.

**Methods:**

This retrospective study enrolled 6,455 fresh embryo transfer cycles at the First Affiliated Hospital of Zhengzhou University between May 1, 2021, and October 31, 2022. The primary outcome is the clinical pregnancy rate (CPR). At the same time, the secondary results are the number of oocytes retrieved, two pronuclei (2PN) rate, blastocyst formation rate, high-quality blastocyst rate, and semen parameters (volume, density, sperm count, forward motility rate, total motility rate, immobility rate, and DNA fragment index (DFI) rate).

**Results:**

In the comparison of ovarian stimulation indicators, no statistically significant differences (P > 0.05) were found in Gn days, endometrial thickness, 2PN rate, metaphase 2 (MII) rate, high-quality embryo rate, and blastocyst formation rate. No significant differences (P>0.05) were found in age, body mass index (BMI), education level, and semen parameters (volume, density, sperm count, forward motility rate, total motility rate, immobility rate, and DFI rate) in these four groups. The multivariate regression model showed that neither the types of vaccines nor the vaccination status of both infertile couples significantly affected clinical pregnancy.

**Discussion:**

The type of vaccine does not appear to have an unfavorable effect on ovarian stimulation, embryo development, semen parameters, and clinical pregnancy.

## Graphical Abstract

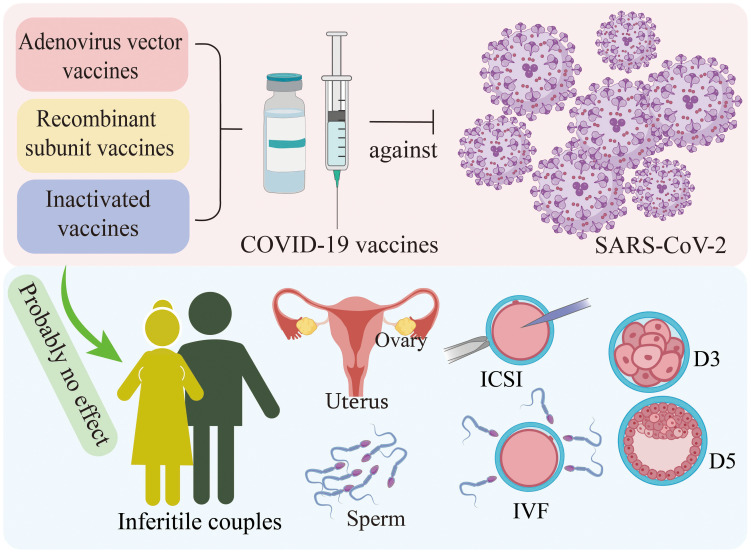

## Introduction

A global pandemic triggered by the rapidly progressing coronavirus disease-2019 (COVID-19) included a consequence for every sector of the world’s economy. Over 750 million cases have been diagnosed globally thus far, and more than 68 million people have been killed as a result (1). With the advent of vaccines against SARS-CoV-2, COVID-19 fatalities and severity have decreased substantially. China has 89.5% of its receiving all doses of the vaccine by 2 February 2023, as opposed to the 46.5% global immunization rate (2). An endorsement from official and professional bodies unequivocally recommends COVID-19 vaccination. The COVID-19 vaccine was developed speedier than any other vaccination or medication, and clinical trials rarely enlist pregnant or of reproductive age participants. Therefore, among women planning to conceive or assisted reproductive technology (ART) -undergoing women’s concerns about their implications for reproductive health, vaccine hesitancy remains high.

Reassuringly, several retrospective studies have confirmed the safety of mRNA vaccines in terms of ovarian function and *in vitro* fertilization (IVF) pregnancy outcome in IVF patients. Bentov et al (3) stated that although anti-COVID IgG antibodies were detected in the follicular fluid of both control and mRNA-vaccinated groups, there was no difference in any of the parameters representing follicular quality. Avraham et al (4) further investigated the effect of mRNA vaccine on IVF outcomes and showed that the vaccine had no effect on ovarian response, fertilization rate, embryo quality and clinical pregnancy rates were not affected. In addition, it was shown that mRNA vaccine also had no effect on embryo implantation and pregnancy outcome in frozen embryo transfer (FET) cycles (5, 6). Safrai et al (7) demonstrated that mRNA Covid-19 vaccine did not affect sperm parameters (8). There are also several studies that further confirmed the safety of C. neoformans mRNA vaccine during IVF (9, 10). The above studies have focused on mRNA vaccine, and in China most of the vaccinations are inactivated. A number of studies exist to prove the safety of inactivated vaccines in fresh or FET cycles without negative effects on ovarian function and pregnancy outcomes (11–15). In addition, inactivated vaccines are also safe in terms of sperm parameters (16, 17).

Adenovirus vector vaccines, recombinant subunit vaccines, and inactivated vaccine products are the three COVID-19 vaccine types currently authorized for application in China. However, studies on the effect of vaccine type and two other vaccines other than inactivated vaccines approved in China on IVF pregnancy outcomes are rare. Requena et al. (18) compared IVF treatment data before and after vaccination and concluded that vaccine type did not adversely affect ovarian function but did not investigate the effect on IVF pregnancy outcomes. Results from a prospective cohort study in China including 750 infertile couples showed no adverse effect of vaccination type on embryo quality and clinical pregnancy rates (19). To complement and confirm the existing findings, this research aimed to investigate whether there are adverse effects of different vaccine types in females and males on reproductive function and clinical pregnancy.

## Materials and methods

### Study population

This retrospective study enrolled 6,455 fresh embryo transfer cycles from infertile couples at the First Affiliated Hospital of Zhengzhou University between May 1, 2021, and October 31, 2022. The target population is people undergoing IVF-ET who have been diagnosed with infertility, which is defined as unsuccessful pregnancy without any contraception for more than one year and with normal sexual life. The infertile population aged 20–40 years were recruited in this study. Exclusion criteria: history of COVID-19 infection, cycles with donor sperm or oocytes, patients undergone controlled ovarian stimulation (COS) ≥ 3 cycles, patients with genetic factors infertility, and couples with severe systemic diseases. Completely vaccinated patients were considered the case group, and unvaccinated patients were defined as the control group. A total of 6,007 cycles were included in this study in accordance with the inclusion and exclusion criteria. The study was approved by the Ethics Committee of the First Affiliated Hospital of Zhengzhou University. All personal information of the subjects was kept confidential.

The relevant data were obtained from the database of the Reproduction Center and included the vaccination status of infertile couples, the date of vaccination, and the type of vaccine. In a further way, there are some primary data, participant characteristics, causes of infertility, indicators of ovarian function, semen parameters, as well as laboratory data, and clinical pregnancy results.

**Figure 1:**
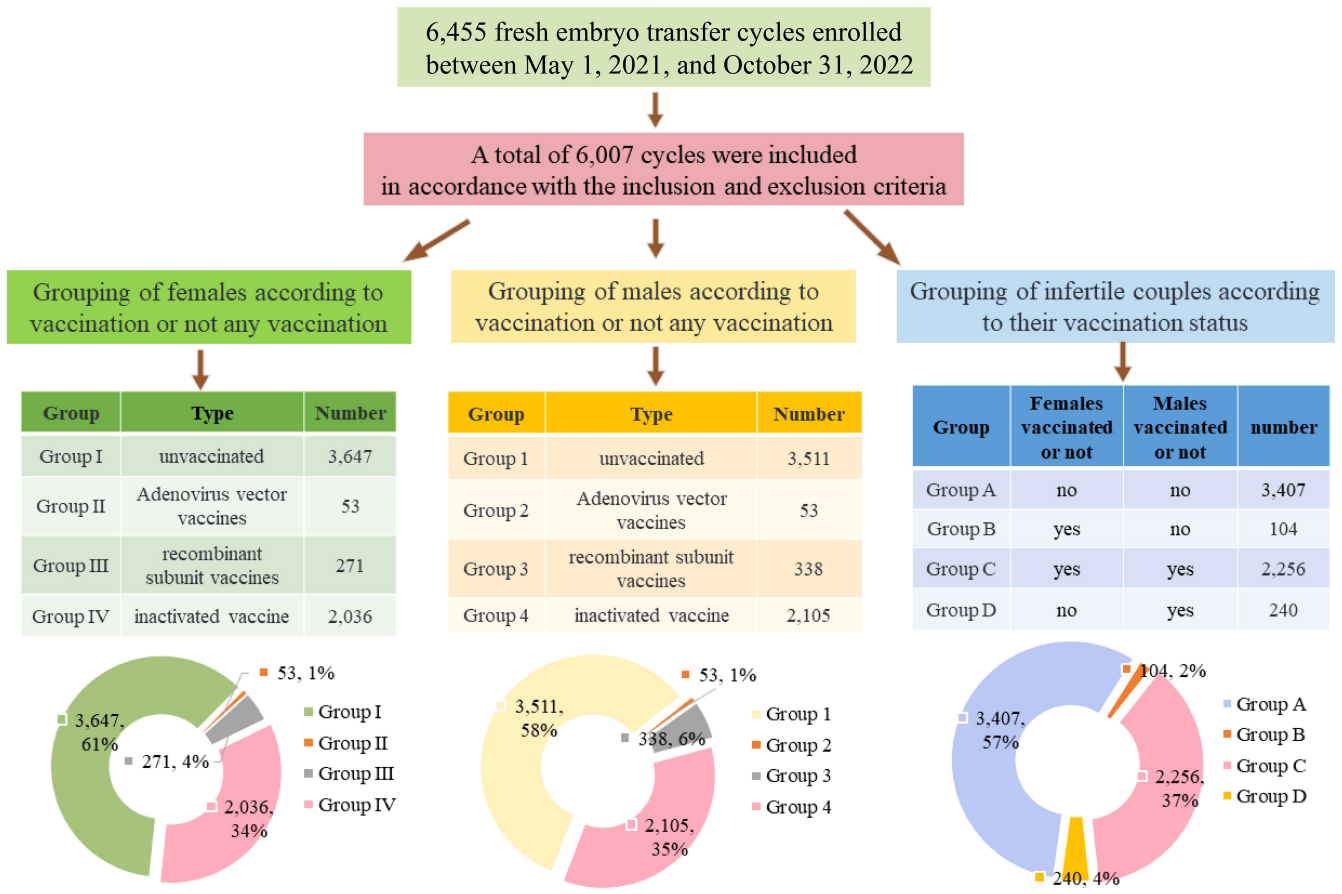
Flow chart and summary table of subgroups and percentage of groups represented by pie charts.

Grouping criteria, displayed in **Figure 1**.

(1) Grouping of females according to vaccination or not any vaccination: Group I, unvaccinated (n = 3,647); Group II, Adenovirus vector vaccines (n = 53); Group III, recombinant subunit vaccines (n = 271); and Group IV, inactivated vaccines (n = 2,036).

(2) Grouping of males according to vaccination or not any vaccination: Group 1, unvaccinated (n = 3,511); Group 2, Adenovirus vector vaccines (n = 53); Group 3, recombinant subunit vaccines (n = 338); and Group 4, inactivated vaccines (n = 2,105).

(3) Grouping of infertile couples according to their vaccination status: Group A, female and male, both unvaccinated (n=3,407); Group B, female vaccinated and male unvaccinated (n=104); Group C, female and male, both vaccinated (n=2,256); Group D, female unvaccinated and male vaccinated (n=240).

### ART protocols

COS was accomplished with gonadotropin-releasing hormone (GnRH) agonist long protocol and antagonist protocol. The COS protocol and Gn initiation dose were determined according to the patient’s age, body mass index (BMI), follicle count, and medical history. The Gn dose during ovulation induction was adjusted according to endocrine levels and follicle development. Details of these protocols were described in the Center’s article (20). When ≥ 2 follicles reached a diameter of ≥ 18 mm or more than 2/3 of follicles reached ≥ 16 mm, ovulation was triggered by Aizer (Merck Serono, Italy) or hCG (Lizhu Medicine). Transvaginal ultrasound-guided oocyte retrieval was conducted approximately 37 hours later. IVF and/or intracytoplasmic sperm injection (ICSI) were performed for fertilization. Depending on the patient’s condition and embryo development, one or two D3/D5 embryo(s) were selected for transfer. Luteal support was initiated on the day of oocyte retrieval with daily transvaginal progesterone (Xenotong, Merck Serono, Switzerland) and oral progesterone (Dupbaston, Abbott, Holland). The serum chorionic gonadotropin (β-hCG) levels > 50 IU/mL at 14 days after the embryo transfer was considered biochemically pregnant. With luteal phase support continued, an abdominal ultrasound was performed on day 35 post-transplant to determine if they were clinically pregnant. Patients with clinical pregnancy were then followed up periodically by telephone to obtain their obstetric results, and this information was recorded in the central archives.

### Outcome measures and definitions

The primary outcome is the clinical pregnancy rate (CPR), and secondary outcomes are the number of oocytes retrieved, two pronuclei (2PN) rate, high-quality blastocyst rate, blastocyst formation rate; and semen parameters (volume, density, sperm count, forward motility rate, total motility rate, immobility rate, and DNA fragment index (DFI) rate). CPR was defined as the number of clinical pregnancies per 100 embryos transferred cycles.

### Statistical analysis

Data were analyzed by SPSS25.0 software. The quantitative data were recorded as mean ± standard deviation (SD). The continuous variables with normal distribution were compared using ANOVA, while those with non-normal distribution were compared using the Wilcoxon rank-sum test. Qualitative data were recorded as frequency and percentage (%), and frequencies were compared using the Chi-square test or Fisher’s exact test. Data were analyzed with the multivariate logistic regression to calculate the adjusted odds ratio (aOR) and 95% confidence interval (CI) for clinical pregnancy to control potential confounders. P < 0.05 was considered statistically significant.

## Results

### Statistical characteristics of the type of vaccination in women and its effect on ovarian function

We encompassed a total of 6,007 fresh embryo transfer (ET) cycles according to the inclusion and exclusion criteria, with a CPR of 57.53% (3,456). The women could be categorized into four groups according to the type of vaccination: 3,647 in the unvaccinated group, 53 in the adenovirus group, 2,036 in the inactivated vaccine group, and 271 in the recombinant vaccine group. There remained no significant differences in age, BMI, education, Anti-Müllerian hormone (AMH), baseline endocrine level, and fertilization type among the four groups (P > 0.05). Still, the differences in infertility factors, number of cycles, number of embryos transferred, and stage of transfer were statistically significant (P < 0.05), as shown in **Table 1**. In the comparison of ovarian stimulation indicators, significant differences (P < 0.05) were found in COS protocol type, Gn initiation dose, total dose, and the number of eggs retrieved. In contrast, no statistically significant differences (P > 0.05) were found in Gn days, endometrial thickness on the day of oocyte retrieval, 2PN rate, metaphase 2 (MII) rate, high-quality embryo rate, and blastocyst formation rate, illustrated in **Table 2**.

**Table 1:** Basic characteristics of the type of vaccination for females.

Variable	Group I (n = 3,647)	Group II (n = 53)	Group III (n = 271)	Group IV (n = 2,036)	P
Age (years)	31.82 ± 4.23	32.19 ± 4.51	31.81 ± 3.70	31.94 ± 4.29	0.711
BMI (kg/m^2^)	23.25 ± 3.26	22.72 ± 3.38	23.22 ± 3.37	23.32 ± 3.36	0.547
Education level					0.707
Graduate degree	163 (4.5%)	2 (3.8%)	13 (4.8%)	91 (4.5%)	
College degree	868 (23.8%)	9 (17.0%)	56 (20.7%)	456 (22.4%)	
Less than college	2616 (71.7%)	42 (79.2%)	202 (74.5%)	1489 (73.1%)	
AFC	12.89 ± 6.61	13.80 ± 6.55	13.42 ± 6.54	12.26 ± 6.39	0.472
AMH (ng/ml)	3.08 ± 2.45	3.16 ± 2.39	3.05 ± 2.14	3.20 ± 2.55	0.371
Basal FSH	7.08 ± 2.77	7.23 ± 2.45	7.04 ± 2.62	6.91 ± 2.27	0.398
Basal LH	5.71 ± 4.28	5.03 ± 3.06	5.75 ± 7.29	5.83 ± 4.61	0.543
Basal E_2_	75.54 ± 282.74	48.38 ± 27.91	44.43 ± 32.20	53.90 ± 133.22	0.181
Basal P	0.54 ± 1.83	0.46 ± 0.57	0.58 ± 1.79	0.51 ± 1.89	0.919
Causes of infertility % (n)					0
PCOS	216 (5.9%)	1 (1.9%)	10 (3.7%)	116 (5.7%)	
Endometriosis	196 (5.4%)	4 (7.5%)	12 (4.4%)	115 (5.6%)	
Ovulatory disorder	179 (4.9%)	6 (11.3%)	4 (1.5%)	78 (3.8%)	
Tubal factors	1599 (43.8%)	19 (35.8%)	127 (46.9%)	924 (45.4%)	
Unexplained infertility	348 (9.5%)	1 (1.9%)	20 (7.4%)	123 (6.0%)	
others	1109 (30.4%)	22 (41.5%)	98 (36.2%)	680 (33.4%)	
The number of cycles					0
1	2856 (78.3%)	46 (86.8%)	233 (86.0%)	1736 (85.3%)	
≥2	791 (21.7%)	7 (13.2%)	38 (14.0%)	300(14.7%)	
Fertilization type					0.237
IVF	2604 (71.4%)	37 (69.8%)	204 (75.3%)	1496 (73.5%)	
ICSI	1043 (28.6%)	16 (30.2%)	67 (24.7%)	540 (26.5%)	
Number of embryos for transfer					0.002
1	1641 (45.0%)	23 (43.4%)	135 (49.8%)	1020 (50.1%)	
2	2006 (55.0%)	30 (56.6%)	136 (50.2%)	1016 (49.9%)	
Embryo stage					0.003
D3	2665 (73.1%)	40 (75.5%)	181 (66.8%)	1386 (68.1%)	
D5	981 (26.9%)	13 (24.5%)	90 (33.2%)	650 (31.9%)	

BMI, Body mass index; AFC, Antral Follicle Count; AMH, Anti-Müllerian hormone; FSH, follicle-stimulating hormone; LH, luteinizing Hormone; PCOS, Polycystic ovarian syndrome; IVF, In Vitro Fertilization; ICSI, Intracytoplasmic sperm injection. Group I, unvaccinated; Group II, Adenovirus vector vaccines; Group III, recombinant subunit vaccines; and Group IV, inactivated vaccines.

**Table 2:** Comparison of ovarian stimulation indices by type of female vaccination.

Variable	Group I (n = 3,647)	Group II (n = 53)	Group III (n = 271)	Group IV (n = 2,036)	P
COS protocols % (n)					0
Early follicular phase long-acting regimen	2947 (80.8%)	46 (86.8%)	241 (88.9%)	1749 (85.9%)	
The classic ultra-long-acting regimen	479 (13.1%)	5 (9.4%)	21 (7.7%)	177 (8.7%)	
Others	221 (6.1%)	2 (3.8%)	9 (3.3%)	110 (5.4%)	
Starting dose of Gn (IU)	194.66 ± 75.48	186.08 ± 67.92	182.52 ± 73.07**γ**	182.44 ± 73.45*	0
Total dose of Gn (IU)	2848.23 ± 1042.24	2707.31 ± 1093.63	2716.61 ± 1051.93	2721.06 ± 1072.63*	0
Days of stimulation	12.78 ± 2.33	12.66 ± 2.74	12.93 ± 2.21	12.92 ± 2.50	0.106
Endometrial thickness (mm)	11.94 ± 2.55	12.55 ± 2.74	12.14 ± 2.55	12.02 ± 2.61	0.465
The number of oocyte retrieval	11.02 ± 5.59	11.15 ± 5.30	11.89 ± 5.69	11.60 ± 5.49*	0.001
2PN rate	0.68 ± 0.20	0.66 ± 0.23	0.67 ± 0.20	0.69 ± 0.19	0.333
MII rate	0.81 ± 0.18	0.78 ± 0.20	0.82 ± 0.17	0.82 ± 0.16	0.253
High-quality embryo rate	0.71 ± 0.27	0.70 ± 0.22	0.70 ± 0.26	0.70 ± 0.26	0.965
Blastocyst formation rate	0.49 ± 0.34	0.47 ± 0.35	0.49 ± 0.35	0.52 ± 0.34	0.106

COS, controlled ovarian stimulation. Group I, unvaccinated; Group II, Adenovirus vector vaccines; Group III, recombinant subunit vaccines; and Group IV, inactivated vaccines.

*Statistically significant difference between Group I and Group IV; **γ** Statistically significant difference between Group I and Group III.

### Statistical characteristics of the type of vaccination in men and its effect on semen parameters

The distribution of male vaccination types was performed as follows, with 3,511 cases in the unvaccinated group, 53 cases in the adenovirus vaccine group, 2,105 cases in the inactivated virus vaccine group, and 338 cases in the recombinant vaccine group. No significant differences (P>0.05) were found in age, BMI, education level, and semen parameters (volume, density, sperm count, forward motility rate, total motility rate, immobility rate, and DFI rate) in these four groups. In the laboratory data, there were no differences in the 2PN rate, MII rate, high-quality embryo rate, and blastocyst formation rate (P>0.05), summarized in **Table 3**.

**Table 3:** Basic characteristics and sperm parameters of the type of vaccination in the males.

Variable	Group 1 (n = 3,511)	Group 2 (n = 53)	Group 3 (n = 338)	Group 4 (n = 2,105)	P
Male age(years)	32.23 ± 5.11	30.98 ± 6.63	31.95 ± 6.62	31.85 ± 4.88	0.170
Male BMI(kg/m^2^)	25.34 ± 4.03	25.12 ± 5.13	25.67 ± 3.57	25.64 ± 3.98	0.356
Education level					0.849
Graduate degree	126 (3.6%)	2 (3.8%)	12 (3.6%)	85 (4.0%)	
College degree	796 (22.7%)	11 (20.8%)	73 (21.6%)	443 (21.0%)	
Less than college	2589 (73.7%)	40 (75.5%)	253 (74.9%)	1577 (74.9%)	
Volume, ml	2.58 ± 1.49	2.60 ± 1.25	2.47 ± 1.04	2.46 ± 1.16	0.223
Sperm concentration, million/ml	35.22 ± 33.64	30.97 ± 20.41	37.96 ± 35.78	38.25 ± 31.07	0.422
Sperm count, million	90.29 ± 109.59	77.13 ± 55.38	78.57 ± 71.57	99.27 ± 98.00	0.436
Progressive motility, %	33.82 ± 11.17	35.82 ± 10.63	34.20 ± 11.40	33.98 ± 11.89	0.232
DFI, %	16.38 ± 11.27	17.27 ± 12.62	16.17 ± 9.65	17.05 ± 11.23	0.231
Immotile, %	55.34 ± 11.75	53.09 ± 9.65	55.19 ± 11.22	55.24 ± 12.45	0.438
Total motility, %	38.69 ± 18.72	38.94 ± 19.83	38.71 ± 18.59	39.87 ± 18.25	0.141
The number of oocyte retrieval	11.00 ± 5.59	11.71 ± 4.77	12.16 ± 5.71**γ**	11.53 ± 5.50*	0
2PN rate	0.68 ± 0.20	0.68 ± 0.21	0.67 ± 0.20	0.69 ± 0.19	0.372
MII rate	0.82 ± 0.18	0.81 ± 0.16	0.82 ± 0.16	0.82 ± 0.17	0.678
High-quality embryo rate	0.71 ± 0.26	0.72 ± 0.25	0.70 ± 0.25	0.71 ± 0.26	0.971
Blastocyst formation rate	0.49 ± 0.34	0.55 ± 0.37	0.50 ± 0.34	0.50 ± 0.37	0.329

BMI, body mass index; DFl, sperm DNA fragment index. Group 1, unvaccinated; Group 2, Adenovirus vector vaccines; Group 3, recombinant subunit vaccines; and Group 4, inactivated vaccines.

*Statistically significant difference between Group 1 and Group 4; **γ** statistically significant difference between Group 1 and Group 3.

### Impact of vaccination status on ART in infertile couples

Given that the aforementioned investigation revealed no significant association between female and male vaccine types on ART outcomes, we further classified infertile couples into four groups depending on whether they were vaccinated or not, without differentiating between vaccine types: female and male both unvaccinated (n=3,407), female vaccinated and male unvaccinated (n=104), female and male both vaccinated (n=2,256), female unvaccinated and male vaccinated (n=240). Ovarian function, semen parameters, and clinical pregnancy were investigated as a result. As indicated in **Table 4**, there were no variations in the infertility factors, number of cycles, number of embryos transferred, or stage of transfer, nor were there changes in the infertile couples’ ages, BMIs, educational levels, or AMH and basal endocrine level in the female. As illustrated by **Table 5**, there were statistically significant differences in the ovarian stimulation parameters for the COS protocol type, total dose of Gn, endometrial thickness, and number of oocytes retrieved (P<0.05), but not for days of stimulation, 2PN rate, MII rate, high-quality embryo rate and blastocyst formation rate (P > 0.05). Moreover, sperm volume, density, sperm count, forward motility rate, and immobility rate did not significantly vary across semen parameters (P > 0.05). There was no discernible difference in the clinical pregnancy rates between the four groups. In the regression analysis of clinical pregnancy, the rude OR and adjusted OR of the four groups were not statistically significant after eradicating confounding variables, displayed in **Table 5**.

**Table 4:** Basic characteristics of the vaccination status of both partners in infertile couples.

Variable	Group A (n=3,407)	Group B (n=104)	Group C (n=2,256)	Group D (n=240)	P
Female Age (years)	31.82 ± 4.23	32.05 ± 4.08	31.92 ± 4.30	31.93 ± 4.24	0.764
Female BMI (kg/m^2^)	23.24 ± 3.26	23.25 ± 3.19	23.45 ± 3.23	23.30 ± 3.37	0.765
Female education level					0.177
Graduate degree	157 (4.6%)	2 (1.9%)	6 (2.5%)	104 (4.6%)	
College degree	804 (23.6%)	29 (27.9%)	64 (26.7%)	492 (21.8%)	
Less than college	2446 (71.8%)	73 (70.2%)	170 (70.8%)	1660 (73.6%)	
Male age(years)	32.23 ± 5.10	31.68 ± 6.40	32.18 ± 5.64	32.67 ± 5.23	0.262
Male BMI(kg/m^2^)	25.32 ± 3.97	25.60 ± 4.03	25.71 ± 4.05	25.35 ± 3.62	0.971
Male education level					0.243
Graduate degree	122 (3.6%)	4 (3.8%)	9 (3.8%)	90 (4.0%)	
College degree	769 (22.6%)	27 (26.0%)	64 (26.7%)	463 (20.5%)	
Less than college	2516 (73.8%)	73 (70.2%)	167 (69.6%)	1703 (75.5%)	
AFC	12.91 ± 6.61	11.47 ± 5.52	11.65 ± 6.67	12.56 ± 6.47	0.340
AMH (ng/ml)	3.07 ± 2.44	3.05 ± 2.27	3.30 ± 2.75	3.19 ± 2.51	0.213
Basal FSH	7.08 ± 2.76	6.90 ± 1.98	7.18 ± 2.91	6.94 ± 2.34	0.530
Basal LH	5.69 ± 4.30	5.16 ± 2.65	5.97 ± 4.07	5.83 ± 5.05	0.326
Basal E2	76.71 ± 290.75	77.78 ± 249.04	59.06 ± 121.14	51.51 ± 115.21	0.092
Basal P	0.54 ± 1.82	0.54 ± 1.92	0.53 ± 1.89	0.52 ± 1.86	0.986
Causes of infertility n (%)					0
PCOS	199 (5.8%)	6 (5.8%)	17 (7.1%)	121 (5.4%)	
Endometriosis	190 (5.6%)	6 (5.8%)	6 (2.5%)	125 (5.5%)	
Ovulatory disorder	166 (4.9%)	3 (2.9%)	13 (5.4%)	85 (3.8%)	
Tubal factors	1504 (44.1%)	33 (37.1%)	95 (39.6%)	1037 (46.0%)	
Unexplained infertility	334 (9.8%)	9 (8.7%)	14 (5.8%)	135 (6.0%)	
others	1014 (29.8%)	47 (45.2%)	95 (39.6%)	753 (33.4%)	
The number of cycles					0
1	2677 (78.6%)	87 (83.7%)	179(74.6%)	1928 (85.5%)	
≥2	730 (21.4%)	17 (16.3%)	61 (25.4%)	328 (14.5%)	
Fertilization type					0.236
IVF	2430 (71.3%)	73(70.2%)	164 (72.5%)	1664 (73.8%)	
ICSI	977 (28.7%)	31 (29.8%)	66 (27.5%)	592 (26.2%)	
Number of embryos for transfer					0.001
1	1523 (44.7%)	48 (46.2%)	118 (49.2%)	1130 (50.1%)	
2	1884 (55.3%)	56 (53.8%)	122 (50.8%)	1126 (49.9%)	
Embryo stage					0
D3	2507 (73.6%)	69 (66.3%)	158 (65.8%)	1538 (68.2%)	
D5	899(26.4%)	35 (33.7%)	82 (34.2%)	718 (31.8%)	

BMI, Body mass index; AFC, Antral Follicle Count; AMH, Anti-Müllerian hormone; FSH, follicle-stimulating hormone; LH, luteinizing Hormone; PCOS, Polycystic ovarian syndrome; IVF, In Vitro Fertilization; ICSI, Intracytoplasmic sperm injection.

**Table 5:** Ovarian stimulation and sperm parameters on the vaccination status of both partners in infertile couples.

Variable	Group A (n=3,407)	Group B (n=104)	Group C (n=2,256)	Group D (n=240)	P
COS protocols % (n)					0
Early follicular phase long-acting regimen	2765 (81.2%)	96 (92.3%)	182 (75.8%)	1940 (86.0%)	
The classic ultra-long-acting regimen	441 (12.9%)	5 (4.8%)	38 (15.8%)	198 (8.8%)	
Others	201 (5.9%)	3 (2.9%)	20 (8.3%)	118 (5.2%)	
Starting dose of Gn (IU)	194.90 ± 75.40	183.89 ± 70.88	191.25 ± 76.72	182.47 ± 73.38*	0
Total dose of Gn (IU)	2857.86 ± 1044.98	2794.11 ± 1003.16	2711.51 ± 994.69**γ**	2716.83 ± 1073.38*	0
Days of stimulation	12.80 ± 2.33	13.09 ± 2.51	12.59 ± 2.38	12.91 ± 2.47	0.075
Endometrial thickness (mm)	11.97 ± 2.56	12.29 ± 2.71	11.49 ± 2.42	12.04 ± 2.60	0.190
The number of oocyte retrieval	11.00 ± 5.60	11.19 ± 5.12	11.39 ± 5.40	11.64 ± 5.53*	0
2PN rate	0.68 ± 0.20	0.69 ± 0.20	0.67 ± 0.20	0.68 ± 0.19	0.556
MII rate	0.81 ± 0.17	0.85 ± 0.15	0.81 ± 0.18	0.82 ± 0.16	0.070
High-quality embryo rate	0.71 ± 0.27	0.70 ± 0.26	0.72 ± 0.27	0.70 ± 0.26	0.735
Blastocyst formation rate	0.49 ± 0.34	0.49 ± 0.33	0.53 ± 0.35	0.50 ± 0.33	0.222
Sperm Parameters					
Volume, ml	2.57 ± 1.43	2.67 ± 2.90	2.54 ± 1.45	2.45 ± 1.11	0.113
Sperm concentration, million/ml	35.34 ± 33.65	37.25 ± 27.97	36.25 ± 33.00	37.10 ± 29.64	0.143
Sperm count, million	90.56 ± 109.69	92.19 ± 88.46	88.51 ± 83.55	93.72 ± 90.56	0.449
Progressive motility, %	33.90 ± 11.02	30.93 ± 15.40	34.71 ± 12.37	33.98 ± 11.74	0.069
DFI	17.59 ± 12.22	18.92 ± 17.21	16.28 ± 9.62	17.08 ± 11.27	0.750
Immotile, %	55.30 ± 11. 64	56.71 ± 15.23	54.02 ± 12.22	55.32 ± 12.24	0.05
Total motility, %	38.85 ± 18.58	39.19 ± 19.95	38.81 ± 19.69	39.57 ± 18.31	0.538

COS, controlled ovarian stimulation; BMl, body mass index; DFl, sperm DNA fragment index.

*Statistically significant difference between Group A and Group C; γ statistically significant difference between Group A and Group D.

### Effect of vaccine types on clinical pregnancy

**Figure 2:**
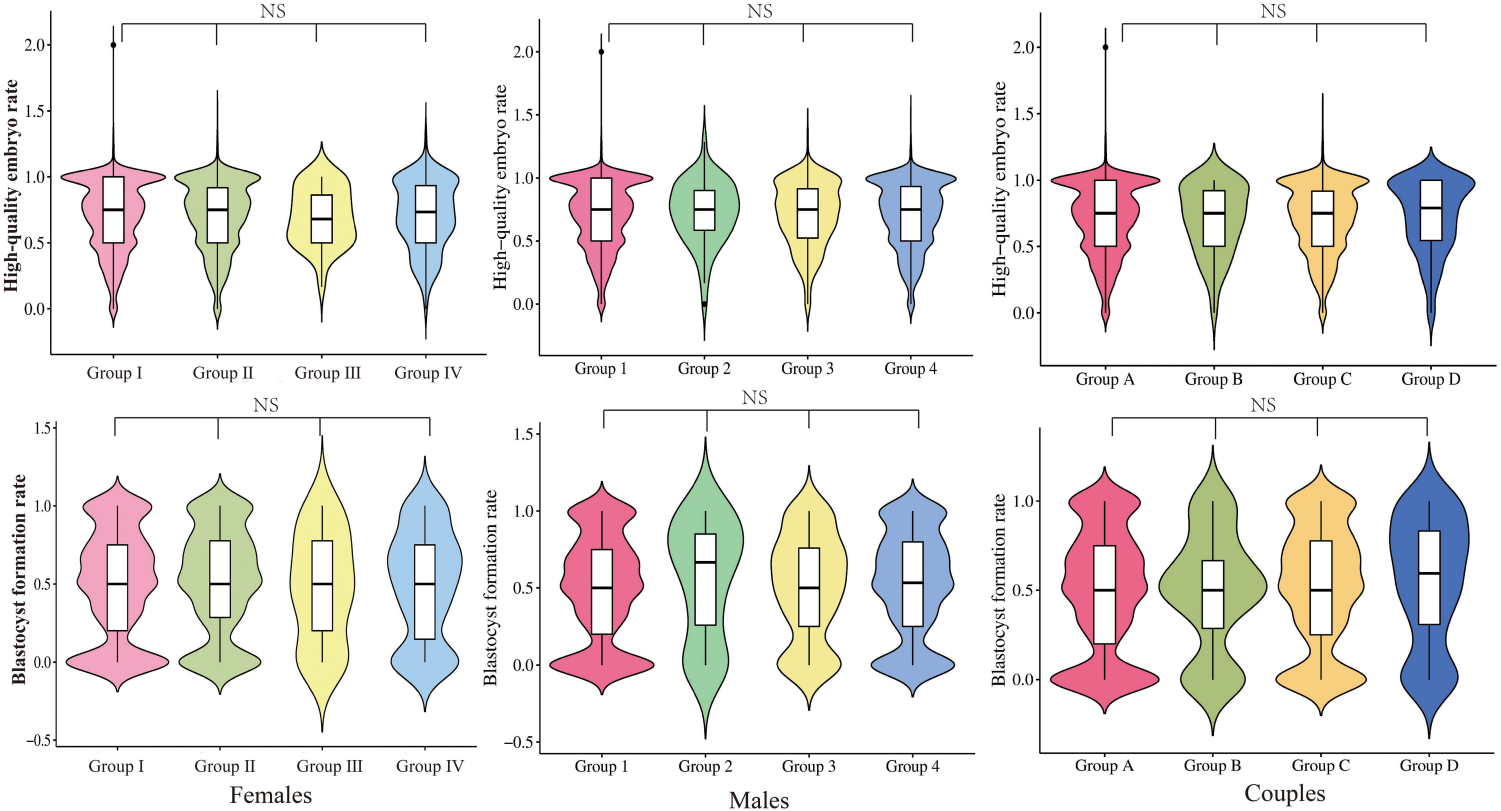
Distribution of high-quality embryo rate and blastocyst formation rate in the group with different vaccine types. NS, No significance.

**Figure 3:**
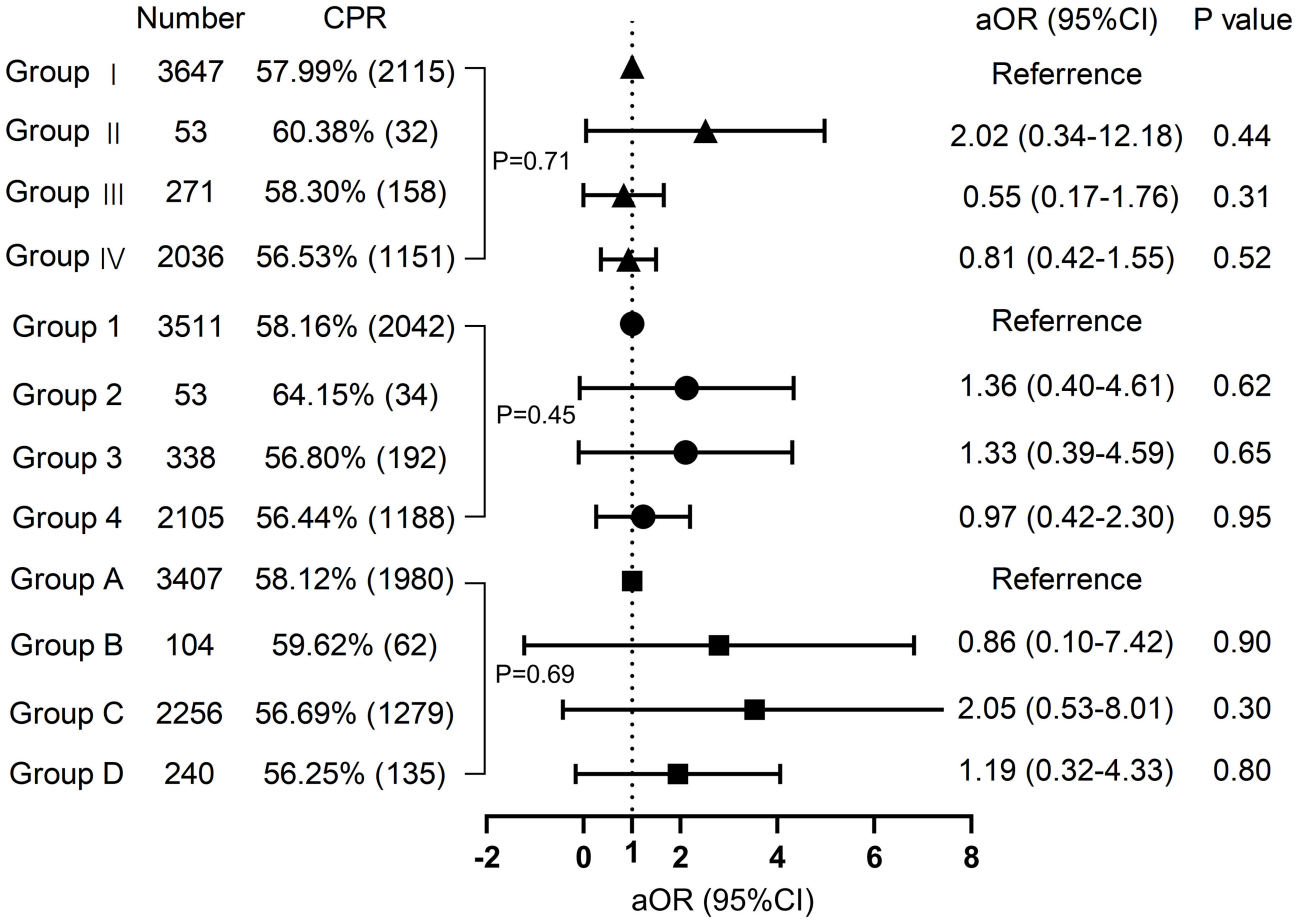
Regression analysis Multivariate regression analysis of groups with different vaccine types on clinical pregnancy. CPR, clinical pregnancy rate.

Neither grouping according to female nor male vaccine type differed in clinical pregnancy rates (58.0% vs. 60.4% vs. 56.5% vs. 58.3%, P=0.708; 58.2% vs. 64.2% vs. 56.4% vs. 56.8% P=0.453, respectively). The type of vaccine given to the female or male partner had no impact on the probability of pregnancy in either group. The high-quality embryo rate and blastocyst formation rate for each of the three subgroups were displayed in violin plots in **Figure 2**, with no significant differences. Subsequently, we proceeded with regression analysis to minimize the effects of confounding variables and generate more precise results. We excluded female age, female BMI, female education level, male age, male BMI, male education level, semen parameters (volume, density, sperm count, forward motility, total motility, immobility, DFI rate), sperm source, sperm retrieval method, sperm storage method, infertility factors, AMH, COS regimen, Gn initiation dose, the total dose of Gn, days of stimulation, endometrial thickness, fertilization type, the number of oocyte retrieval, embryo transferred stage, the number of embryos transferred, and other potential confounding factors. As portrayed in **Figure 3**, vaccine type remained unaffected on clinical pregnancy odds when categorized according to the kind of vaccination received by the female partner after adjusting for confounding factors (aOR=2.02, 95% CI=0. 34–5.18; aOR=0.81, 95% CI=0.42–1.55; aOR=0.55, 95% CI=0.17–1.76, respectively). With regard to the male vaccination type group, there was similarly no influence of vaccine type on the probability of clinical pregnancy (aOR=1.36, 95%CI=0.40–4.61; aOR=0.97, 95% CI=0.41–2.30; aOR=1.33, 95%CI=0.39–4.58, respectively).

## Discussion

To our knowledge, the present study is the first to simultaneously focus on the effects of vaccine type on ovarian stimulation, semen parameters, and clinical pregnancy in both females and males. Our findings suggested that the type of vaccine had almost no effect on ovarian stimulation and no effect at all on semen parameters and clinical pregnancy. On top of that, the same results were observed with and without vaccination in both females and males. Taken together, these results recommend that regardless of the type of COVID-19 vaccine administered prior to performing IVF, it does not influence the IVF outcomes.

The global outbreak of COVID-19 has created an urgent need for vaccination, initiating a global wave of COVID-19 vaccination. The severity of SARS-CoV-2 virus infection in pregnant women is heightened compared to non-pregnant women, with an increased risk of requiring mechanical ventilation, ICU admission, and death (21). It may lead to adverse neonatal outcomes such as preterm delivery (22). Hence, women of reproductive age need to be vaccinated against COVID-19 before pregnancy, which is effective in reducing the incidence of severe disease. However, the low vaccination rate among infertile women attending reproductive centers may be owing to the lack of evidence on whether the COVID-19 vaccine has a detrimental impact on assisted reproductive outcomes.

Numerous studies have reported no harm from COVID-19 vaccination in women of reproductive age or during pregnancy (23–25), as well as no adverse effects on semen parameters (26, 27). However, relatively few studies have been conducted on the impact on infertile couples who are to undergo assisted reproductive treatment. Avraham et al. (4) demonstrated no effect of the COVID-19 mRNA vaccine on ovarian response and CPR in fresh or frozen cycles. Aharon (5) proved that the COVID-19 mRNA vaccine does not influence ovarian stimulation, clinical pregnancy, and miscarriage rates in single euploid frozen-thawed embryo transfer cycles. Likewise, Aizer et al. (6) analyzed 672 FET cycles. They determined that IVF implantation rates, CPR, and ongoing pregnancy rates after SARS-CoV-2 infection or vaccination were similar to those of uninfected or unvaccinated individuals. One study identified little if any, impact of the vaccine on IVF outcome when examining anti-nucleotide (anti-N) and anti-synuclein (anti-S) antibodies in follicular fluid and blood samples on the oocyte retrieval day (10). Similarly, another study found no statistically significant differences in FET cycles between SARS-CoV-2 vaccine seropositive, infected seropositive, and seronegative women in terms of the incidence of implantation (8). In contrast, Youngster et al. (28) arrived at the opposite result, with previous SARS-CoV-2 infection leading to a reduced chance of clinical pregnancy in FET (aOR 0.325, 95% CI 0.106–0.998; P = 0.05) and more significantly transfer within 60 days of infection (OR 0.072, 95% CI 0.012–0.450; P = 0.005). Transcriptome analyses have implicated COVID-19 disease, leading to altered endometrial gene expression, enriched for immune and inflammatory aspects (29). Furthermore, no significant adverse effect on follicular function was demonstrated (3). Most of these studies addressing the impact of COVID-19 vaccines on IVF outcomes have focused on mRNA vaccines.

In China, a number of studies have also emerged on the effect of COVID-19 vaccines on IVF, but almost all have been on inactivated vaccines. Wu et al. (11) explored that inactivated vaccination in fresh embryo transfer hardly affected ovarian stimulation, embryo development, and CPR. The team subsequently discovered that inactivated vaccines in the FET cycle did not impair live birth and neonatal outcomes (12). Another research team similarly documented the harmlessness of inactivated vaccines in fresh or FET cycles for IVF treatment while not being affected by the time interval between vaccination and IVF initiation (14, 15). In counterpoint, shi et al. (13) emphasized that inactivated vaccination within 60 days prior to IVF entails a significant reduction in CPR and proposed that IVF should be delayed until at least two months after vaccination.

However, findings on other types of COVID-19 vaccines are scarce, and inactivated vaccines are not representative of all vaccine types. One study explored the effect of vaccine type on IVF (19), and the results of this prospective cohort study, including 750 infertile couples, revealed that the style and interval of vaccination of the female partner had no adverse effect on embryo quality and clinical pregnancy rates. However, there were some problems that the encompassing population included both fresh and frozen cycles, which may have been detrimental to the reliability of the results. In addition, the study did not include the adenovirus-vaccinated population. Yin et al. (30) investigated that adenovirus vaccine and inactivated vaccines did not affect follicular development, embryo quality, and clinical pregnancy rates. Chen et al. (31) suggested that inactivated vaccines and recombinant vaccines had no effect on embryo quality and clinical pregnancy rates, but observed that inactivated vaccines may impair oocyte maturation and fertilization rates. Specific information on related studies is shown in **Table 6**. Compared with the above study, the present study set a more comprehensive grouping of vaccine types, including adenovirus vector vaccines, recombinant subunit vaccines and inactivated vaccines. Moreover, the present study also investigated that male vaccination types and cross-grouping of male and female vaccination status had no effect on IVF outcomes. In particular, no Chinese research has examined the impact of male vaccination types on semen parameters and clinical pregnancy, and the present study adds to this, finding no deleterious effects. This study confirms that fresh cycles after vaccination with the COVID-19 vaccines, regardless of vaccine types, have little or no adverse effect on ovarian stimulation, sperm parameters, embryo quality and clinical pregnancy. The hesitation to confront concerns about the harmful effects of vaccines is not supported by the evidence from this study.

**Table 6:** Studies on the effect of COVID-19 vaccines on IVF procedures.

Article	Country and publication year	Embryo transfer type	No. of cycles included	Adenovirus vector vaccines	Recombinant subunit vaccines	Inactivated vaccines	mRNA vaccines	Ovarian function	Sperm parameters	Embryo quality	Clinical pregnancy rates	Live birth rate	Others
Dong et al. (19)	China, 2022	Fresh or Frozen	735	/	√ n=11	√ n=163	/	/	/	NS	NS	/	/
Requena et al. (18).	Spain, 2023	Fresh or Frozen	510	√ n=69	/	/	√ n=441	NS	/	/	/	/	/
Chen et al. (31)	China, 2023	Fresh	536	/	√ n=44	√ n=223	/	Lower oocyte maturation	NS	NS	NS	/	Decreased fertilization rate
Yin et al. (30)	China, 2023	Fresh	2505	√ n=68	/	√ n=278	/	NS	/	NS	NS	/	/
Avraham et al. (4)	Israel, 2022	Fresh or Frozen	400	/	/	/	√ n=200	NS	/	NS	NS	/	/
Aharon et al. (5)	America, 2022	Frozen	1205	/	/	/	√ n=222	NS	/	NS	NS	/	/
Aizer et al. (6)	Israel, 2022	Frozen	672	/	/	/	√ n=220	/	/	NS	NS	/	/
Odeh-Natour et al. (10)	Israel, 2022	Fresh	59	/	/	/	√ n=37	NS	/	NS	NS	/	/
Bentov et al. (3)	Israel, 2021	/	32	/	/	/	√ n=9	NS	/	/	/	/	/
Albeitawi et al. (32)	Jordan, 2022	Fresh	151	/	/	/	√ n=66	NS	/	NS	NS	/	/
Safrai et al. (7)	Israel, 2022	/	72	/	/	/	√ n=72	/	NS	/	/	/	/
Karavani et al. (33)	Israel, 2022	/	58	/	/	/	√ n=58	/	NS	/	/	/	/
Wu et al. (11)	China, 2022	Fresh	1583	/	/	√ n=240	/	NS	/	NS	NS	/	/
Cao et al. (12)	China, 2022	Frozen	2091	/	/	√ n=502	/	NS	/	NS	NS	NS	Ectopic, birth height and weight NS
Huang et al. (15)	China, 2022	Fresh	2185	/	/	√ n=150	/	NS	/	NS	NS	/	/
Huang et al. (14)	China, 2023	Frozen	1210	/	/	√ n=387	/	NS	/	NS	NS	NS	/
Xia et al. (16)	China, 2022	Fresh or Frozen	260	/	/	√ n=105	/	/	NS	NS	NS	/	/
Zhu et al. (17)	China, 2022	/	43	/	/	√ n=43	/	/	NS	/	/	/	/

Studies have shown a higher risk of morbidity and mortality in pregnant women who are unvaccinated with the COVID-19 vaccine (34). COVID-19 vaccination during pregnancy is very safe and effective, a formidable tool to forestall maternal and neonatal morbidity. It has been demonstrated not to increase the risk of fetal congenital anomalies, miscarriage, preterm delivery, small for gestational age infants or other adverse perinatal outcomes (35–38). IgM (39, 40), SARS-CoV-2 Spike mRNA or protein (41), and pathological changes (42) were not detected in the placenta or umbilical cord blood of vaccinated pregnant women, which more reliably confirm the safety of the COVID-19 vaccine, are consistent with the findings mentioned above. Furthermore, the incidence of local and systemic immune responses after vaccination in pregnant women is more consistent with the general population (43–45). Compared with vaccinated women of childbearing age in the non-pregnant state, there were no changes in the titers of anti-Spike, anti-RBD (receptor binding domain of the Spike protein), SARS-CoV-2 neutralizing antibodies, and Spike-induced production of IFNγ by total and central memory CD4^+^ and CD8^+^ T cells (44, 46). Moreover, the IgG produced by indirect vaccination during pregnancy provides prevention and protection to the fetus through the placental barrier (47–49). COVID-19 vaccination during pregnancy is safe for infants, which is recommended for people planning pregnancy, during pregnancy and breastfeeding (50).

The safety and efficacy of COVID-19 vaccines has been demonstrated in the general population by some high-level evidence of a reduction in the incidence of severe or critical illness (51). For special populations such as planned pregnancies and pregnant women, the evidence for safety is as described above (38). Older people are more likely to be infected with COVID-19 than younger people and are more likely to develop serious illnesses when infected (52). The available evidence suggests that the COVID-19 vaccine is effective and well tolerated in the elderly population and that the benefits of vaccination far outweigh any potential risks (53). However, the effectiveness of standard COVID-19 vaccination regimens in immunocompromised patients is unknown. A meta-analysis of patients using TNF alpha inhibitors did not effectively produce antibodies after COVID-19 vaccination (54). Unique vaccination programs and further studies should be designed for this population. The long-term side effects of the COVID-19 vaccine remain unclear, with some studies raising the possibility of cross‐reaction, with one study suggesting that individuals with strong immune responses are more likely to develop autoimmune diseases than others when vaccinated with nucleic acid vaccines (55, 56). This will require future studies to be conducted to determine whether there are any long-term side effects associated with COVID-19 vaccination. In addition, the longevity of vaccination is unknown and vaccine trials are necessary to clarify this. In conclusion, the benefits of COVID-19 vaccination far outweigh the risks, and strict adherence to health guidelines for active vaccination is an important strategy.

With the considerable risk of SARS-CoV-2 infection to pregnant women and their infants and the safety and efficacy of COVID-19 vaccination proven by numerous studies, vaccination of women of childbearing age and pregnant women for protection is essential and infertile women about to undergo IVF are included. This study demonstrates that different types of COVID-19 vaccines have no measurable negative impact on IVF pregnancy outcomes, providing evidence to dispel further the misconception that the COVID-19 vaccine impairs female or male fertility. Available evidence does not suggest any association between COVID-19 vaccination and adverse outcomes of IVF treatment. Prior to initiating IVF-ET, it is necessary to ensure that the patient completes the vaccination program and encourage vaccination with any of the available COVID-19 vaccines. This may help to increase vaccination rates, reduce the spread of COVID-19 virus, and protect infertile patients and their families from the risk of infection. Since the type of vaccine has no negative impact on IVF outcomes, vaccination policies may be more flexible.

The chief merit of this research is that it complements the existing evidence on whether COVID-19 vaccination affects IVF outcomes by exploring the impact of different vaccination types on clinical pregnancy. In addition, the breakdown of vaccination status in infertile couples further confirms the safety of COVID-19 vaccination. The multifactorial analysis controlled for the effects of confounding factors and increased the robustness and reliability of the results. Inevitably, certain limitations exist. First, we did not further inquire into the impact of vaccine type on live births and neonatal outcomes due to the limited duration of follow-up. Second, information on COVID-19 vaccination was obtained from patient self-reports and may be at risk of misclassification. Additionally, fewer people in the adenovirus vaccination group of 53 cases were included in this study because of the lower number of individuals who received adenovirus vaccination. Studies with larger sample sizes are warranted to confirm the findings of this research. Ultimately, this research is a single-center case-control study, and multicenter investigations are necessary to augment the generalizability of the results.

## Conclusions

The type of vaccine and vaccination status in infertile couples do not appear to have an unfavorable effect on ovarian stimulation, embryo development, semen parameters, and clinical pregnancy.

## Data availability statement

The raw data supporting the conclusions of this article will be made available by the authors, without undue reservation.

## Ethics statement

Written informed consent was obtained from the individual(s) for the publication of any potentially identifiable images or data included in this article. This retrospective study was approved by the Ethics Committee of the First Affiliated Hospital of Zhengzhou University.

## Author contributions

SW: Conceptualization, Data curation, Formal Analysis, Investigation, Methodology, Visualization, Writing – original draft. NW: Data curation, Resources, Writing – review & editing. GY: Investigation, Resources, Writing – review & editing. YS: Conceptualization, Project administration, Resources, Supervision, Writing – review & editing. LQ: Conceptualization, Investigation, Methodology, Project administration, Resources, Supervision, Writing – review & editing.
